# A Remarkable Man

**Published:** 1880-12

**Authors:** 


### A REMARKABLE MAN.

Many of the leading journals of this country have recently printed notices of the remarkable longevity of the oldest living lawyer in actual practice, Asgill Gibbs, Esq., of Rochester, N. Y. Mr. Gibbs is now in his ninety-fourth year, and for seventy years has never been absent from his office for a single day on account of illness. He is to-day in the enjoyment of perfect health and in possession of all his faculties. This wonderful longevity and freedom from illness are the direct result of a course of living which this journal has advocated for more than a quarter of a century, and we dwell upon this particular instance of the results of careful living because all the facts are personally known to us, Mr. Gibbs being the father of the editor of this journal.

The secret of this long exemption from any serious disease and of this green old age is an open one. It is simply the avoidance in daily life of such things as all the world knows impair the health and strength of mankind and bring on decay. Mr. Gibbs has never used tobacco in any form, and, as for intoxicating liquors, he is ignorant of their taste. His diet has always been ample but simple. Fond of the pleasures of the table, he enjoys them in moderation. An active and laborious life has been sweetened and prolonged by a rigid enforcement of the homely but golden rule, "Do not fret."

The incalculable benefits of this abstemious and careful life have not been confined to the subject of this sketch. His children have inherited sound constitutions, and have preserved them by the practice of the simple rules of living, the good effects of which have always been before their eyes. Tobacco and strong drinks are banished as by instinct from their homes, and "health by good living," not *high* living or fast living, is a family motto. The result has been that in a family of nine—the parents, six sons and one daughter—there was no serious illness and no death in forty years, and during seventy years but two deaths. Of the seven survivors not one possesses a physical weakness or infirmity.

We have never before made reference to family affairs in the pages of the JOURNAL, thinking that to do so, under ordinary circumstances, would not be in good taste. But if the above facts are the means of convincing a single one of the many who contend that "a *moderate* indulgence in the use of liquor and tobacco can do no harm," that it is far safer not to indulge at all, we shall not regret having published this article.

